# Glycosides from *Stevia rebaudiana* Bertoni Possess Insulin-Mimetic and Antioxidant Activities in Rat Cardiac Fibroblasts

**DOI:** 10.1155/2017/3724545

**Published:** 2017-08-30

**Authors:** Cecilia Prata, Laura Zambonin, Benedetta Rizzo, Tullia Maraldi, Cristina Angeloni, Francesco Vieceli Dalla Sega, Diana Fiorentini, Silvana Hrelia

**Affiliations:** ^1^Department of Pharmacy and Biotechnology, Alma Mater Studiorum, University of Bologna, Via Irnerio, No. 48, 40126 Bologna, Italy; ^2^Department for Life Quality Studies, Alma Mater Studiorum, University of Bologna, Corso d'Augusto, No. 237, 47921 Rimini, Italy; ^3^Department of Surgery, Medicine, Dentistry and Morphological Sciences, University of Modena and Reggio Emilia, Policlinico, Via del Pozzo, No. 71, 41124 Modena, Italy; ^4^School of Pharmacy, University of Camerino, Via Gentile III da Varano, 62032 Camerino, Italy; ^5^Department of Medical Sciences, University of Ferrara, Via Luigi Borsari, No. 46, 44121 Ferrara, Italy

## Abstract

*Stevia rebaudiana* Bertoni is a shrub having a high content of sweet diterpenoid glycosides in its leaves, mainly stevioside and rebaudioside A, which are used as noncaloric, natural sweeteners. The aim of this study was to deepen the knowledge about the insulin-mimetic effect exerted by four different mixtures of steviol glycosides, rich in stevioside and rebaudioside A, in neonatal rat cardiac fibroblasts. The potential antioxidant activity of these steviol glycosides was also assessed, as oxidative stress is associated with diabetes. Likewise the insulin effect, steviol glycosides caused an increase in glucose uptake into rat fibroblasts by activating the PI3K/Akt pathway, thus inducing Glut4 translocation to the plasma membrane. The presence of S961, an insulin antagonist, completely abolished these effects, allowing to hypothesize that steviol glycosides could act as ligands of the same receptor engaged by insulin. Moreover, steviol glycosides counteracted oxidative stress by increasing reduced glutathione intracellular levels and upregulating expression and activity of the two antioxidant enzymes superoxide dismutase and catalase. The present work unravels the insulin-mimetic effect and the antioxidant property exerted by steviol glycosides, suggesting their potential beneficial role in the cotreatment of diabetes and in health maintenance.

## 1. Introduction

*Stevia rebaudiana* Bertoni is a shrub belonging to the Asteraceae family, native to Paraguay and Brazil, and has been now cultivated in many parts of the world [1–3]. Due to the high content of sweet diterpenoid glycosides in its leaves, the “sweet herb of Paraguay” has been used for many years in South America for sweetening food products and in traditional medicine, particularly as a natural control for diabetes [4]. *Stevia rebaudiana* Bertoni has attracted scientific interest for its potential use as noncaloric [5, 6] and noncariogenic [7, 8] sweetener and also for its multifaceted benefits on human health [9] and therapeutic properties [10, 11]. Several studies indeed suggest that *Stevia* has antihyperglycaemic, antihypertensive, antitumour, antidiarrheal, diuretic, anti-inflammatory, and immune-modulatory effects [12]. Owing to these characteristics, leaf extracts rich in steviol glycosides, such as stevioside and rebaudioside A, have been authorized as commercial sweeteners and food additives [13], as the safety of high-purity steviol glycosides has been extensively reviewed in the published literature and by national and international food safety agencies [14].

Since the prevalence of diabetes is rapidly rising all over the world, the identification of nontoxic, natural compounds from a plant origin, able to mimic the insulin action, appears to be of great interest and importance, according to the last WHO expert committee recommendations [15]. In this context, the use of *Stevia* extracts as natural sweeteners is notably useful in restricting or controlling caloric intake in the diet or as a substitute for sucrose in the treatment of diabetes. The mechanisms by which *Stevia* leaves and its steviol glycosides exert a marked antidiabetic effect have been intensively studied [16]. Some reports account for an increased insulin secretion by stevioside [17] or rebaudioside A [18]; other demonstrated that stevioside increases insulin sensitivity in rodent models [19], inducing antihyperglycaemic effects on diabetic rats [20] and on diabetic subjects [21, 22]; moreover, insulin-mimetic properties of steviol and stevioside have been reported in L6 and 3T3L1 cell lines [23]. Furthermore, as oxidative stress is among the features correlated with diabetic condition, the antioxidant activity recently attributed to *Stevia* further highlights the potential synergic beneficial effect of this plant [24–27].

**Figure 1:**
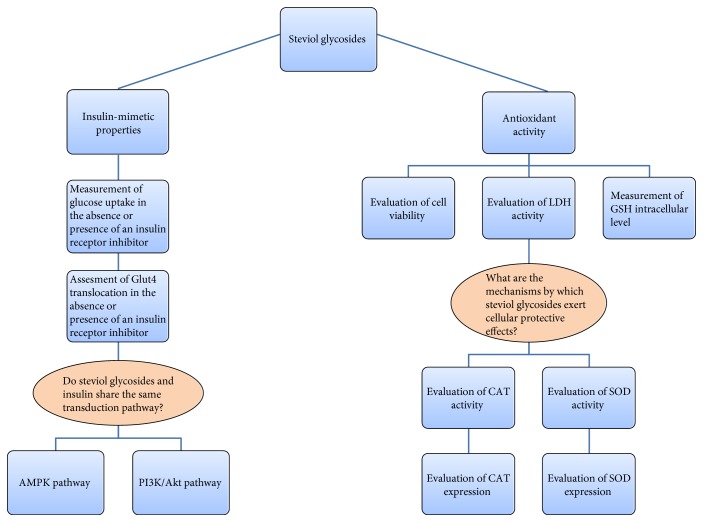
Graphical representation of the experimental design.

We previously demonstrated that steviol glycosides exert a marked insulin-like effect on glucose transport activity in cancer cell lines [28]. The aim of this study, graphically reported in Figure 1, was to investigate the molecular mechanisms underpinning the insulin-mimetic effect showed by steviol glycosides in a nontransformed cell system. Moreover, the potential antioxidant property of steviol glycosides was also examined. Neonatal rat cardiac fibroblasts were chosen, as they are fully involved in high glucose-induced cardiac fibrosis, a pathological consequence of diabetes, leading to cardiac dysfunction [29]. Cardiac fibroblasts, indeed, express the insulin receptor (IR) [30], the insulin growth factor-like 1 and its receptor (IGF-1/IGF-1R) [31], and the insulin-responsive glucose transporter (Glut4) [32].

## 2. Materials and Methods

### 2.1. Chemicals and Reagents

Dulbecco's modified Eagle's medium (DMEM), foetal bovine serum (FBS), penicillin, streptomycin, 3-(4,5-dimethylthiazol-2-yl)-2,5-diphenyltetrazolium bromide (MTT), insulin, 2-deoxy-glucose (DOG), phloretin, DAPI, RIPA lysis buffer, 10% SDS solution, mammalian protease inhibitor mixture, phosphatase inhibitor cocktail PhosSTOP (Roche™), hydrogen peroxide, Tris-HCl, sodium pyruvate, NADH, monochlorobimane (MCB), SOD assay kit, primary antibody to *β*-actin, bovine serum albumin (BSA), and all other chemicals were purchased from Sigma-Aldrich. S961 insulin receptor antagonist was from Phoenix Peptide. 2-Deoxy-D-[2,6-3H]-glucose and Ultima Gold MV scintillation cocktail were from PerkinElmer. Anti-Glut4 (sc-1606) antibody was obtained from Santa Cruz Biotechnology. Rabbit anti-goat IgG (H+L) secondary antibody Alexa Fluor® 488 conjugate (A11078) was purchased from Life Technologies. Primary antibodies against phospho-Akt (Ser473) (#4058), Akt (#9272), phospho-AMPK*α* (Thr172) (#2535), AMPK*α* (#4058), and IGF-1 receptor *β* (IGF-1R) (#3027) and horseradish peroxidase-conjugated secondary antibodies anti-rabbit (#7074) and anti-mouse (#7076) were purchased from Cell Signaling Technologies. Primary antibodies anti-phospho-PI3 kinase p85 pTyr458/p55 pTyr199 (#PA5-17387) and anti-phospho-IGF-1R pTyr1165+pTyr1166 (#PA5-35452) were from Thermo Scientific. Anti-PI3 kinase (#06-195) antibody was purchased from Millipore. DC™ Protein Assay, 4–20% Mini-PROTEAN® TGX™ Precast Gels, Precision Plus Protein™ Unstained Standards, and Clarity™ Western ECL Substrate were from Bio-Rad. Catalase Assay Kit was from Cayman Chemical. RNA-to-cDNA Conversion Kit was from Applied Biosystems. RNA Miniprep Kit was from Agilent Technologies. RT-PCR primers for superoxide dismutase 1 (SOD1), catalase (CAT), *β*-2-microglobulin (B2M), and actin (ACT) were manufactured from Sigma-Aldrich.

Four different mixtures of steviol glycosides, differing in their relative content, were provided by *Stevia* extraction companies and used in this study.

REB A 97 (R97) was from Pure Circle SDN BHD (Negeri Sembilan, Malaysia) and, according to the certificate of analysis, contains >97% rebaudioside A and <3% other steviol glycosides.

RA60 (R60) was from HYET Sweet B.V. (Breda, the Netherlands) and, according to the certificate of analysis, contains 95.48% total steviol glycosides, of which 63.43% are rebaudioside A, 22.85% are stevioside, 8.21% are rebaudioside C, 0.73% are dulcoside A, and 0.26% are other steviol glycosides.

SG95 (SG) was from Pure Circle SDN BHD (Negeri Sembilan, Malaysia) and, according to the certificate of analysis, contains >95.0% total steviol glycosides, of which >50.0% are rebaudioside A and at least 25% are stevioside.

RA50 (TRU) was from Eridania Italia SpA. (Bologna, Italy) and contains 95.0% total steviol glycosides, of which at least 50% are rebaudioside A and 25% are stevioside and 20% other steviol glycosides are not analytically quantified.

**Figure 2:**
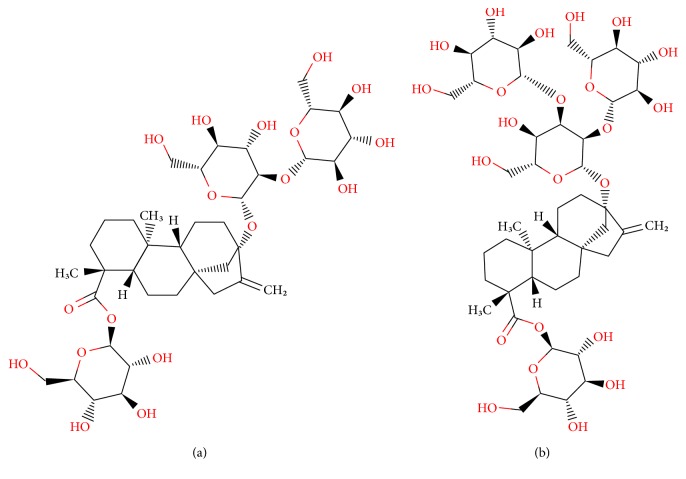
Chemical structures of stevioside (a) and rebaudioside A (b).

The structures of the main steviol glycosides, stevioside and rebaudioside A, are reported in Figure 2.

### 2.2. Cell Culture

Neonatal Sprague-Dawley rat cardiac fibroblasts were a kind gift of Dr. Antonello Lorenzini (Department of Biomedical and Neuromotor Sciences, Alma Mater Studiorum—University of Bologna, Italy). Cells were grown in Dulbecco's modified Eagle's medium (DMEM) supplemented with 10% (*v*/*v*) foetal bovine serum (FBS), 2 mM glutamine, 100 U/mL penicillin, and 100 *μ*g/mL streptomycin, at 37°C in a humidified atmosphere maintained at 5% CO_2_.

### 2.3. Cell Viability

Cell viability was evaluated by the MTT assay. Neonatal rat cardiac fibroblasts were treated with increasing concentrations of R97, R60, SG, and TRU (0.5–5 mg/mL). After 24 h, cells were stressed or not with 100 *μ*M H_2_O_2_ for 30 min and then incubated with 0.5 mg/mL MTT for 4 h at 37°C. To dissolve the blue-violet formazan salt crystals formed, a solubilisation solution (10% SDS, 0.01 M HCl) was added and the plates were incubated overnight in a humidified atmosphere (37°C, 5% CO_2_) to ensure complete lysis. The absorbance at 570 nm was measured using a multiwell plate reader (Wallac Victor2, PerkinElmer).

### 2.4. Glucose Transport Assay

Glucose transport assay was performed as described in [28, 33]. Cells were incubated or not with R97, R60, SG, or TRU (1 mg/mL) for 1 h or with insulin (100 nM) for 30 minutes, in the presence (or not) of S961 (10 nM) for 90 min. Then, they were washed in PBS and treated for 10 min at 37°C with a mixture of 2-deoxy-D-[2,6-3H] glucose (0.8 *μ*Ci/assay) and 1.0 mM unlabeled glucose analogue (DOG), under conditions where the uptake was linear at least for 20 min. The transport was stopped by adding phloretin (final concentration 0.3 mM), a potent inhibitor of glucose transport activity. Cells were washed twice with PBS, detached and resuspended with 1 mL cold PBS, and added to Ultima Gold MV scintillation cocktail (PerkinElmer). Radioactivity was measured by liquid scintillation counting (Tri-Carb® liquid scintillation analyzer, PerkinElmer).

### 2.5. Immunofluorescence

Neonatal rat cardiac fibroblasts, grown on coverslips, were treated with R97 or R60 (1 mg/mL) for 1 hour or with insulin (100 nM) for 30 min in the presence or absence of 10 nM S961 for 90 min and then fixed in 3% (*w*/*v*) paraformaldehyde for 15 min. Cells were washed twice with PBS, blocked with 1% (*w*/*v*) PBS/BSA for 1 hour, and then incubated for 1 hour with 20 *μ*g/mL of goat anti-Glut4 antibody raised against a peptide within an extracellular domain of the glucose transporter protein. Subsequently, cells were treated for 1 hour with fluorescent FITC-conjugated rabbit anti-goat IgG in the dark, nuclei were stained with DAPI, and coverslips were mounted on slides. Confocal imaging was performed by a Nikon A1 confocal laser scanning microscope (Nikon Instruments, Japan).

### 2.6. Immunoblotting Analysis

After treatments with R97, R60, SG, or TRU (1 mg/mL) for 1 hour or with insulin (100 nM) for 30 min, rat cardiac fibroblasts were washed with ice-cold PBS and lysed with RIPA buffer containing mammalian protease and phosphatase inhibitor mixtures. Protein concentration was measured by Bio-Rad DC Protein Assay (Bio-Rad Laboratories). Proteins were separated on 4–20% SDS-PAGE Mini-PROTEAN TGX Precast Gels using a Mini-PROTEAN II apparatus (Bio-Rad Laboratories) and electrophoretically transferred to the nitrocellulose membrane (Hybond-C; GE Healthcare). To avoid nonspecific binding, membranes were incubated in blocking buffer containing 5% (*w*/*v*) albumin in Tris-buffered saline (TBS)/Tween and probed overnight at 4°C with primary antibodies (anti-phospho-IGF-1R, anti-IGF-1R, anti-phospho-PI3K, anti-PI3K, anti-phospho-Akt, anti-Akt, anti-phospho-AMPK, anti-AMPK, or anti-*β*-actin as internal normalizers). Nitrocellulose membranes were then washed with TBS/Tween and incubated with horseradish peroxidase-labelled secondary antibodies in 5% albumin TBS/Tween at room temperature for 1 hour and successively washed with TBS/Tween. Chemiluminescence detection was performed using Clarity Western ECL Substrate (Bio-Rad Laboratories). Bands were acquired with a CCD imager (ChemiDoc™ MP System, Bio-Rad) and analyzed by using Image Lab analysis software (Bio-Rad).

### 2.7. Lactate Dehydrogenase Assay

Rat fibroblasts were incubated with R97, R60, SG, or TRU (1 mg/mL) for 24 hours and then stressed using 100 *μ*M H_2_O_2_ for 30 min. Lactate dehydrogenase (LDH) release from cells into the culture medium was detected by monitoring LDH activity through a spectrophotometric assay based on the reduction of pyruvate to lactate coupled with NADH oxidation to NAD^+^. The decrease in absorbance at 340 nm resulting from the oxidation of NADH was monitored at 37°C in a Varian Cary 50 Spectrophotometer.

### 2.8. Determination of Glutathione (GSH) Levels

Reduced GSH levels were determined by the monochlorobimane (MCB) fluorometric assay as previously reported [34, 35]. Briefly, rat cardiac fibroblasts were incubated for 24 hours with R97, R60, SG, or TRU (1 mg/mL) and exposed or not to 100 *μ*M H_2_O_2_ for 30 min. After treatment, the culture medium was removed and the cells were washed with cold PBS and incubated for 30 min at 37°C with 50 *μ*M MCB in PBS. The strong fluorescence of the GSH-MCB adduct was measured in a multiwell plate reader (Wallac Victor2, PerkinElmer). Excitation wavelength was 355 nm and emission wavelength was 460 nm.

### 2.9. Superoxide Dismutase Assay

Rat cardiac fibroblasts were incubated for 24 hours with R97, R60, SG, or TRU (1 mg/mL), and then, superoxide dismutase (SOD1) activity was measured using the SOD Assay Kit provided by Sigma-Aldrich and following the manufacturer's instructions. SOD Assay Kit-WST is a colorimetric indirect assay method based on Dojindo's highly water-soluble tetrazolium salt WST-1 (2-(4-Iodophenyl)-3-(4-nitrophenyl)-5-(2,4-disulfophenyl)-2H-tetrazolium, monosodium salt) that is reduced by superoxide anion to a stable water-soluble formazan with high molar absorptivity. The absorbance at 440 nm is proportional to the amount of superoxide anion generated from xanthine oxidase provided in the kit; SOD activity was quantified as inhibition activity by spectrophotometrically following the decrease in the color development at 440 nm in a multiwell plate reader (Wallac Victor2, PerkinElmer).

### 2.10. Catalase Assay

Rat cardiac fibroblasts were incubated for 24 hours with R97, R60, SG, or TRU (1 mg/mL), and then, catalase (CAT) activity was quantified by Cayman's Catalase Assay Kit following the manufacturer's protocol. This kit, according to the method of Johansson et al. [34], exploits the peroxidative activity of CAT, and it is based on the oxidation, catalyzed by CAT, of methanol (the electron donor) in the presence of an optimal concentration of H_2_O_2_. The produced formaldehyde was measured spectrophotometrically with the chromogen Purpald® (4-amino-3-hydrazino-5-mercapto-1,2,4-triazole), which reacts with aldehydes producing purple color. The absorbance was monitored at 540 nm in a multiwell plate reader (Wallac Victor2, PerkinElmer). CAT activity was calculated from the amount of formaldehyde produced in the assay.

### 2.11. Analysis of mRNA Expression by RT-PCR

Total RNA was extracted from fibroblasts using a commercially available kit (Absolutely RNA Miniprep Kit, Agilent Technologies), according to the manufacturer's instructions. The quantification of RNA was performed using a NanoVue Spectrophotometer (GE Healthcare) by analyzing at A260/A280 and A260/A230. mRNA was reverse-transcribed into cDNA starting from 1 *μ*g of total RNA using a high-capacity RNA-to-cDNA Conversion Kit (Applied Biosystems). The subsequent PCR was carried out in a total volume of 20 *μ*L containing 2 *μ*L of cDNA, 10 *μ*L SsoAdvanced™ Universal SYBR Green Supermix (Bio-Rad Laboratories), and 1 *μ*L (500 nM) of each primer. The primers used are as follows: CAT (forward) 5′-CAAGTTCCATTACAAGACTGAC-3′ and (reverse) 5′-TAAATGGGAAGGTTTCTGC-3′, SOD (forward) 5′-AATGTGTCCATTGAAGATCG-3′ and (reverse) 5′-CACATAGGGAATGTTTATTGGG-3′, *β*-actin (forward) 5′-AAGACCTCTATGCCAACAC-3′ and (reverse) 5′-TGATCTTCATGGTGCTAGG-3′, and *β*2-microglobulin (forward) 5′-ACTGGTCTTTCTACATCCTG-3′ and (reverse) 5′-AGATGATTCAGAGCTCCATAG-3′.

All primers were produced by Sigma-Aldrich. *β*-Actin and *β*2-microglobulin were used as reference genes. cDNA amplification was started by activating the polymerase for 30 s at 95°C, followed by 40 cycles of 5 s at 95°C and 30 s at 60°C. A melt curve was run to ensure quality control and the generation of a single product. Normalized expression levels were calculated relative to control cells according to the 2^−ΔΔCT^ method.

### 2.12. Statistical Analysis

Results are expressed as means ± SD. Differences among the means were determined by Bonferroni's multiple comparison test following one-way ANOVA and were considered significant at *p* < 0.05.

## 3. Results

### 3.1. Effect of Steviol Glycosides on Cell Viability

Rat cardiac fibroblasts were treated with increasing concentrations of R97, R60, SG, or TRU (0.5–5 mg/mL) for 24 hours to investigate their direct effect on cell integrity/damage. There was no observed decrease in the ability of cardiac fibroblasts to reduce MTT following exposure to the four different mixtures up to 5 mg/mL, indicating the absence of toxicity in this range of concentrations (data not shown). These results are in agreement with previously reported data obtained in different cell types [28].

### 3.2. Effect of Different Steviol Glycosides on Glucose Transport Activity and on Glut4 Translocation to the Plasma Membrane

**Figure 3:**
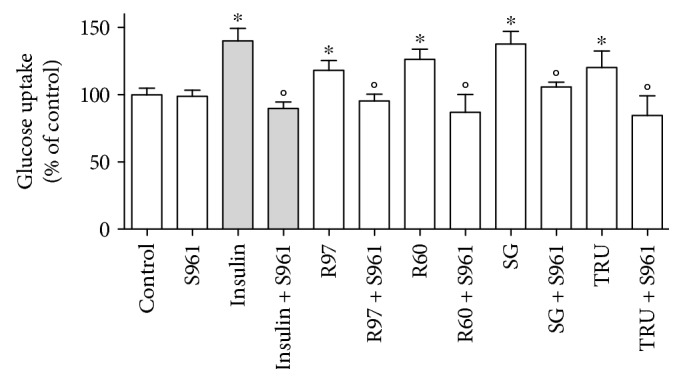
Effect of different steviol glycoside mixtures or insulin on glucose transport activity in rat cardiac fibroblasts in the presence or absence of the antagonist of the insulin receptor S961. Cells were treated with R97, R60, SG, or TRU (1 mg/mL) for 1 hour or with insulin (100 nM) for 30 min, in the presence or absence of 10 nM S961 for 90 min. Glucose uptake was assayed as described in the experimental procedure section. Results are expressed as means ± SD of three independent experiments, each performed in triplicate. Statistical analysis was performed by Bonferroni's multiple comparison test following one-way ANOVA. ^∗^*p* < 0.05 significantly different from control cells; °*p* < 0.05 significantly different from the corresponding cells not treated with S961.

Neonatal rat cardiac fibroblasts were incubated for 1 hour with R97, R60, SG, or TRU (1 mg/mL) or with 100 nM insulin and then assayed for glucose transport activity.  Figure 3() shows that all mixtures were able to significantly enhance glucose uptake at a similar extent, and, interestingly, the increase in glucose transport activity was comparable to that induced by insulin exposure. To deeper study the mechanism of glucose transport induced by steviol glycosides, S961, a 43-amino-acid biosynthetic peptide antagonist of insulin receptor [36], was used. Cell preincubation with 10 nM S961 completely abolished the increase in glucose transport due to steviol glycosides or insulin treatment (Figure 3()).

**Figure 4:**
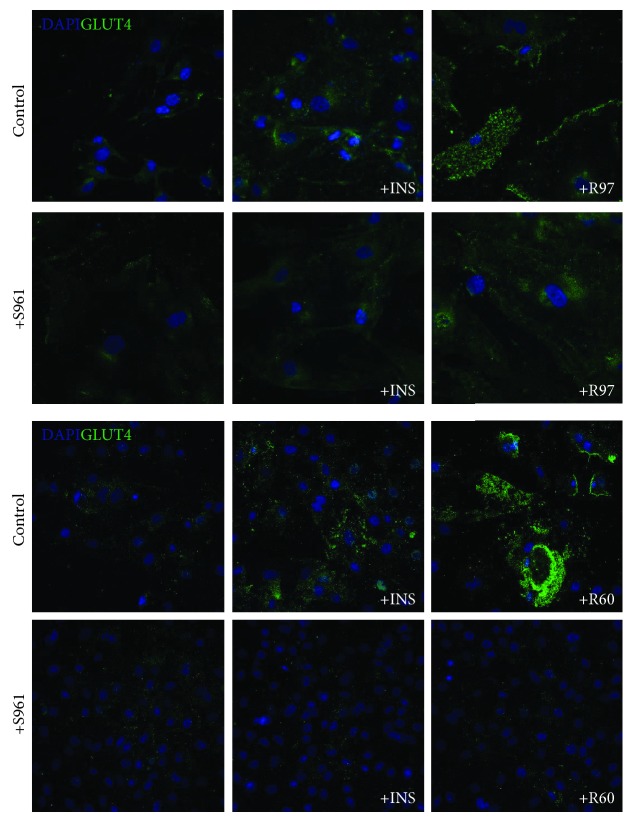
Effect of R97, R60, or insulin on Glut4 translocation to the plasma membrane in rat cardiac fibroblasts in the presence or absence of the antagonist of the insulin receptor S961. Cells were treated with R97 or R60 (1 mg/mL) for 1 hour or with insulin (100 nM) for 30 min in the presence or absence of 10 nM S961 for 90 min. Glut4 translocation to the plasma membrane was detected using immunofluorescence staining with anti-Glut4 antibody and DAPI nuclear staining as a counterstain. Images were acquired by the Nikon A1 confocal laser scanning microscope (Nikon Instruments, Japan). The results are representative of two independent experiments.

It is well known that insulin induces the translocation of Glut4 from cytosolic storage vesicles to the plasma membrane, thereby enhancing glucose transport activity [37]. Therefore, the translocation of Glut4 to the plasma membrane following treatment with steviol glycosides or insulin was assessed by utilizing a Glut4-specific antibody targeting an epitope in the extracellular domain near the N-terminus and visualized through confocal microscopy.  Figure 4 shows the effect of two representative mixtures, R97 and R60, in comparison with that of insulin, in the presence or absence of the antagonist S961. Results indicate that R97 and R60 are able to induce Glut4 translocation to the plasma membrane with comparable efficiency and that their effect is similar to that obtained by insulin stimulation. Moreover, cell pretreatment with S961 counteracts the observed Glut4 translocation both in insulin-stimulated cells and in steviol glycoside-treated cells. Experiments with SG and TRU revealed similar results, indicating that all the samples share the ability to modulate Glut4 translocation with the same efficiency (data not shown). These results are in accordance with those obtained in the evaluation of glucose transport activity.

### 3.3. Effect of Steviol Glycosides on PI3K/Akt and AMPK Pathways

**Figure 5:**
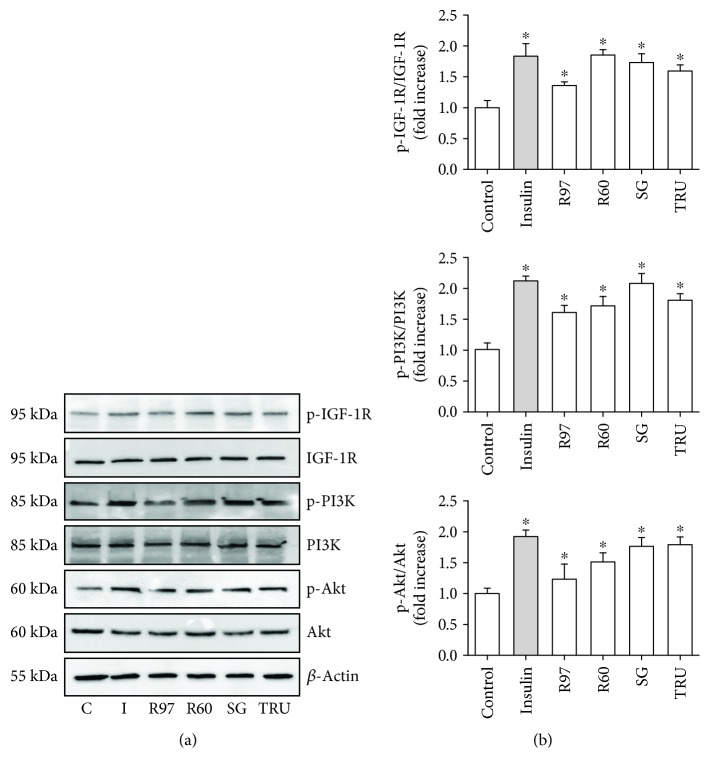
Effect of different steviol glycoside mixtures or insulin on the PI3K/Akt pathway in rat fibroblasts. (a) Rat cardiac fibroblasts treated with R97, R60, SG, or TRU (1 mg/mL) for 1 hour or with insulin (100 nM) for 30 min were lysed with RIPA buffer. Cell lysates were electrophoresed and immunoblotted with the indicated antibodies, as described in the experimental procedure section. Actin detection was used as a load control. Immunoblots are representative of three independent experiments. (b) Densitometric analysis of protein phosphorylation status is expressed as phospho-protein/total protein and reported as folds to control. ^∗^*p* < 0.05 significantly different from control cells.

To assess whether steviol glycosides exert their effect on glucose transport activity through the IR or IGF-1R, the activation of the PI3K/Akt pathway, which mediates the intracellular signaling triggered by the IR family through IR substrates [38], was investigated. The effects of R97, R60, SG, or TRU on activation/phosphorylation of IGF-1R, PI3K, and Akt were examined by immunoblotting, and the results are shown in Figure 5. Cell treatment with 1 mg/mL of the four different mixtures or with 100 nM insulin caused a significant increase in the phosphorylated isoforms of IGF-1R, PI3K, and Akt, indicating a common pathway in insulin and steviol glycoside signaling mechanisms.

**Figure 6:**
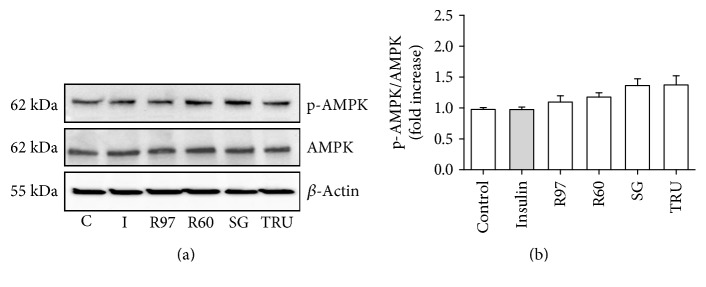
Effects of different steviol glycoside mixtures or insulin on AMPK phosphorylation level in rat cardiac fibroblasts. (a) Rat cardiac fibroblasts treated with R97, R60, SG, or TRU (1 mg/mL) for 1 hour or with insulin (100 nM) for 30 min were lysed with RIPA buffer. Cell lysates were electrophoresed and immunoblotted with anti-AMPK and anti-phospho-AMPK, as described in the experimental procedure section. Actin detection was used as a control. Immunoblots are representative of three independent experiments. (b) Densitometric analysis of AMPK phosphorylation level is expressed as phospho-AMPK/total AMPK and reported as folds to control.

The activation of the metabolic pathway regulated by AMP-activated protein kinase (AMPK) is also known to increase Glut4 translocation and glucose uptake in the heart, especially during physical activity [39]. To test the possible contribution of the AMPK-dependent metabolic pathway to the glucose transport stimulation induced by steviol glycosides, the phosphorylation level of AMPK was investigated by the immunoblotting technique. Results in Figure 6 indicate that treatment with R97, R60, SG, TRU, or insulin did not alter the phosphorylation level of AMPK in these experimental conditions.

### 3.4. Evaluation of the Potential Antioxidant Activity of Steviol Glycosides in Rat Cardiac Fibroblasts

**Figure 7:**
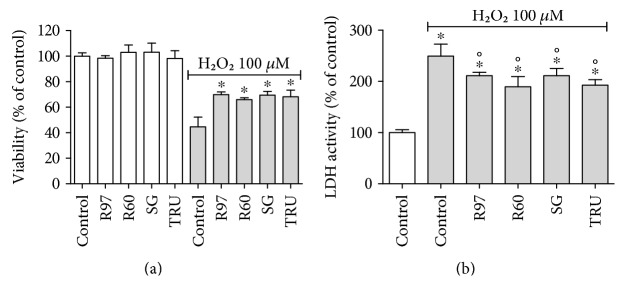
Effect of different steviol glycoside mixtures on rat cardiac fibroblast viability/proliferation and lactate dehydrogenase (LDH) activity. Rat cardiac fibroblasts were treated with R97, R60, SG, or TRU (1 mg/mL) for 24 hours; then, cells were stressed (or not) with 100 *μ*M H_2_O_2_ for 30 min. Cell viability/proliferation was evaluated by the MTT assay (a) or by the LDH assay (b). Results are expressed as means ± SD of three independent experiments. Statistical analysis was performed by Bonferroni's multiple comparison test following one-way ANOVA. ^∗^*p* < 0.05 with respect to the control; °*p* < 0.05 with respect to control cells treated with H_2_O_2_.

In order to evaluate a potential antioxidant/protective effect exerted by the four mixtures under study, fibroblasts were treated with R97, R60, SG, or TRU for 24 hours, stressed (or not) by exogenous addition of H_2_O_2_, and tested for viability by the MTT assay (Figure 7(a)). The viability of cells pretreated with steviol glycosides was significantly increased compared to that of H_2_O_2_-treated cells, evidencing their potential protective role against oxidative stress. Moreover, lactate dehydrogenase (LDH) activity in the culture medium was quantified under stressed conditions, as a nonspecific marker of cell damage. As shown in Figure 7(b), fibroblast pretreatment with steviol glycosides was able to significantly counteract the LDH release from the cells.

The effect of steviol glycosides was also evaluated on intracellular glutathione (GSH) level and on the activities of cytosolic superoxide dismutase (SOD1) and catalase (CAT).

**Figure 8:**
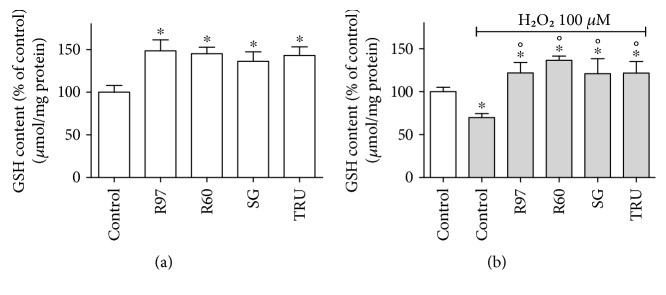
Effect of different steviol glycoside mixtures on intracellular GSH levels in rat cardiac fibroblasts. (a) Rat cardiac fibroblasts were treated with R97, R60, SG, or TRU (1 mg/mL) for 24 hours, and then, intracellular GSH levels were measured using the fluorescence probe MCB as described in the experimental procedure section. (b) Upon pretreatment with steviol glycosides, rat fibroblasts were exposed to 100 *μ*M H_2_O_2_ for 30 min and then assayed for intracellular GSH levels. Results are expressed as means ± SD of four independent experiments. Data were analyzed by one-way analysis of variance (ANOVA) followed by Bonferroni's test. ^∗^*p* < 0.05 significantly different from the control; °*p* < 0.05 significantly different from control cells treated with H_2_O_2_.

Rat cardiac fibroblasts were pretreated with R97, R60, SG, or TRU for 24 hours, and then, GSH intracellular levels were measured.  Figure 8(a) shows that basal GSH levels were significantly higher in cells pretreated with steviol glycosides in respect to controls. When fibroblasts were exposed to H_2_O_2_ (Figure 8(b)), a significant decrease in basal GSH levels occurred, whereas cell pretreatment with steviol glycosides significantly counteracted this GSH depletion, allowing the maintenance of high GSH levels.

**Figure 9:**
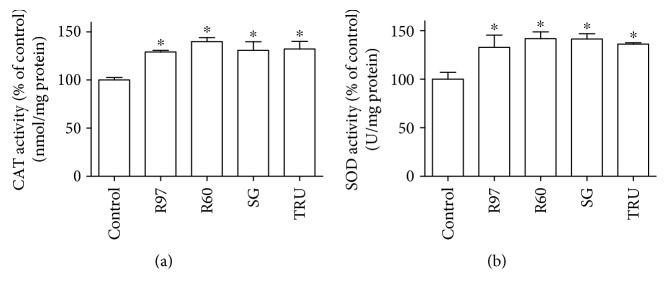
Effect of different steviol glycoside mixtures on catalase (CAT) and superoxide dismutase (SOD1) activities in rat cardiac fibroblasts. Rat cardiac fibroblasts were treated with R97, R60, SG, or TRU (1 mg/mL) for 24 hours and then lysed and assayed for CAT (a) and SOD1 (b), as described in the experimental procedure section. Results are expressed as means ± SD of four independent experiments. Data were analyzed by one-way analysis of variance (ANOVA) followed by Bonferroni's test. ^∗^*p* < 0.05 significantly different from the control.

SOD and CAT are considered primary antioxidant enzymes, directly involved in the removal of ROS.  Figure 9 shows that CAT (Figure 9(a)) and SOD1 (Figure 9(b)) activities significantly increase in rat fibroblasts treated with steviol glycosides for 24 hours compared to controls.

**Figure 10:**
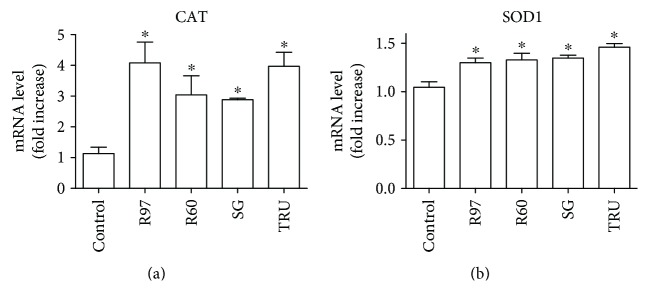
Effect of different steviol glycoside mixtures on catalase (CAT) and superoxide dismutase (SOD1) mRNA levels in rat cardiac fibroblasts. Cells were treated with R97, R60, SG, or TRU (1 mg/mL) for 24 hours, and after RNA extraction, the level of CAT mRNA (a) and SOD1 mRNA (b) was assayed according to the experimental procedure section. Each bar represents the mean ± SD of three independent experiments. Data were analyzed by one-way analysis of variance (ANOVA) followed by Bonferroni's test. ^∗^*p* < 0.05 with respect to the control.

In order to verify whether steviol glycoside was able to induce an upregulation of these antioxidant enzymes also at the transcriptional level, fibroblasts treated with 1 mg/mL of R97, R60, SG, or TRU for 24 h were lysed and analyzed for SOD1 and CAT cytosolic mRNA content by reverse transcriptase-polymerase chain reaction (RT-PCR).  Figure 10(a) shows that an increase in CAT mRNA amount of about 3-4 folds with respect to that of control is observed upon cell treatment with the steviol glycosides, and also, SOD1 mRNA content (Figure 10(b)) is significantly increased, although at a minor extent.

## 4. Discussion

*Stevia rebaudiana* Bertoni and its glycosides are known not only as natural, noncaloric sweeteners but also for their antihypertensive, anti-inflammatory, immune-modulatory, and antihyperglycaemic effects [9]. In particular, the hypoglycaemic, antidiabetic, and insulin-like properties of steviol glycosides have been investigated in 3T3-L1 adipocytes [40], in L6 myoblasts [21], and in cancer-derived cell lines [28].

In this paper, we focused on the insulin-mimetic and antioxidant activities of steviol glycosides in neonatal rat cardiac fibroblasts. We demonstrated that both insulin and steviol glycosides are able to increase glucose entry into the cells by activating the PI3K/Akt—but not the AMPK—pathway, thus inducing Glut4 translocation to the plasma membrane. We also showed that steviol glycosides counteract oxidative stress increasing GSH levels and SOD and CAT expressions and activities.

Fibroblasts express the insulin receptor (IR) [41]; furthermore, it has been reported that the IGF-1/IGF-1R system is crucial in the modulation of rat cardiac fibroblast growth [31]. Both IGF-1R and IR receptors belong to the same family, which includes the IR in two isoforms, IR-A and IR-B, forming homo- or heterodimers, and in cells expressing both IR and IGF-1R, also IGF-1R/IR hybrids [42]. Insulin binds with high affinity both to IR and IGF-1R [41]. Indeed, as reported in Figure 4, IGF-1R is expressed in rat cardiac fibroblasts and it becomes phosphorylated upon cell treatment with insulin. Owing to its physiological role played in cell growth, IGF-1R is also involved in glucose homeostasis through the PI3K/Akt pathway, considered the predominant downstream signaling pathway for the IR family [38].

Given the membership of the two receptors to the same family and the signal proceeding through a common downstream pathway, we did not discriminate between either of receptor engagement upon insulin or steviol glycoside stimulation.

The increased phosphorylation level of the receptor IGF-1R observed upon fibroblast treatment with insulin or the four different mixtures indicates that both insulin and steviol glycosides are able to activate the PI3K/Akt pathways. Through this pathway, they significantly increase the glucose uptake by intensifying the trafficking of Glut4 isoform from intracellular stores to the plasma membrane, thus demonstrating the ability of steviol glycosides to mimic the insulin action.

Similar results were obtained in diabetes-induced myotubes and adipocytes by Bhasker and coworkers [23] who observed an increased level of the Glut4 protein and glucose transport, together with an enhanced transcription of Glut4 mRNA upon steviol or stevioside treatment, although stevioside achieved its efficacy at a higher concentration.

Furthermore, to confirm the possibility that stevioside and rebaudioside A could act as agonists of the same receptor engaged by insulin, we used the insulin antagonist S961. This molecule is a single-chain peptide reported to be a full IR antagonist [36] and stated to be active with a similar potency in cells expressing mostly IR or IGF-1R, probably through a hybrid receptor IR/IGF-1R-mediated response [43]. These authors described also a mixed agonist/antagonist activity for S961, depending on the cell type and concentration, and when using S961 as an IR antagonist *in vitro*, a concentration starting from 10 nM or above is recommended. In rat fibroblasts used in our study, 10 nM S961 behaved as insulin antagonist and completely abolished both the insulin and the steviol glycoside effects on glucose transport activity, confirming the ability of these molecules to trigger the PI3K/Akt signaling pathway through the same insulin receptor.

Insulin and contraction are the two key *stimuli* that acutely regulate Glut4 recruitment to the plasma membrane of the heart, and cardiac fibroblast contractility is one of the characteristics of myocardial remodeling [44]. These two *stimuli* initiate distinct signaling mechanisms, but both lead to increased Glut 4 translocation and glucose uptake [39]. The contraction signaling pathway proceeds through AMPK, and although the downstream targets mediating Glut4 vesicular trafficking have not been completely identified, it is known that AMPK stimulation of glucose transport does not involve downstream activation of the PI3K/Akt pathway [45]. Since crosstalk between these two pathways cannot be excluded [46] and neonatal fibroblasts are isolated from rat heart, the potential involvement of the AMPK pathway in the observed enhanced glucose transport exerted by steviol glycosides was investigated, but the results reported in Figure 6 ruled out this possibility.

These data support the hypothesis that steviol glycosides are able to mimic the insulin action, explaining, at least in part, the antidiabetic effect ascribed to *Stevia rebaudiana* Bertoni.

However, other mechanisms may be responsible for this effect; in fact, increased oxidative stress caused by prolonged hyperglycaemia has also been reported to play a major role in the pathogenesis of this disease [47]. It is well known that the permanent hyperglycaemia characterizing diabetes causes glucose autoxidation and glycation of proteins [48], which thereby depletes the antioxidant defence system thus promoting free radical generation. Moreover, advanced glycation end products and insulin both activate NAD(P)H oxidases, leading to further ROS production [49]. To this regard, many authors have reported a direct antioxidant activity of extracts from leaves of *Stevia rebaudiana* Bertoni, owing to the presence of alkaloids, flavonoids, and polyphenols [9]; nevertheless, biological systems may operate via multiple mechanisms [50]. Indeed, some phytochemicals exert beneficial effects by triggering adaptive stress response signaling pathways, resulting in the increased production of cytoprotective proteins, including antioxidant and phase 2 enzymes, heat shock proteins, growth factors, and protein generally involved in the regulation of cellular redox homeostasis and energy metabolism [51, 52].

Our data demonstrate that steviol glycosides exhibit a significant protective role against H_2_O_2_-induced damage, supporting fibroblast viability and inhibiting LDH release from the cells. According to Bender and coworker [24], no direct antioxidant activity is expected from purified stevioside or rebaudioside A, since steviol glycosides can hardly be absorbed by cells *in vitro*. In order to clarify the mechanism responsible for this observed protective effect, we investigated whether steviol glycosides, by activating the IR/IGF-1R pathway, can modulate endogenous enzymatic and nonenzymatic systems involved in the antioxidant equipment at protein and/or transcriptional level. It has been reported that the stimulation of IGF-1R upon IGF-1 treatment diminishes the oxidative stress and apoptotic effect of high dose of H_2_O_2_ on human umbilical vascular endothelial cells, and it was demonstrated that this protective effect is mediated by the PI3K/Akt pathway [53]. Interestingly, a significant increase in the basal GSH intracellular level together with a significant enhancement in SOD and CAT expressions and activities was observed in rat fibroblasts upon steviol glycoside treatment. GSH plays a central role in coordinating the antioxidant defence processes in the body, and the treatment with steviol glycosides maintained its high level also after the stressful action of H_2_O_2_. Since a decline in the activity of SOD and CAT antioxidant enzymes together with a reduction in GSH levels was described in diabetic animals [26, 27], our results suggest that these antioxidant effects can contribute to the antidiabetic property exhibited by *Stevia rebaudiana* Bertoni.

## 5. Conclusions

**Figure 11:**
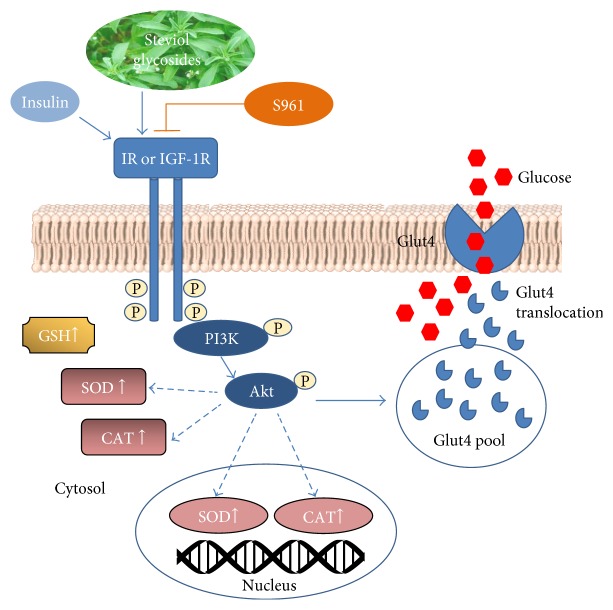
Insulin-mimetic and antioxidant activities of steviol glycoside mixtures. Steviol glycosides are able to act as ligands of the insulin receptor (IR or IGF-1R), triggering the PI3K/Akt pathway. The insulin receptor antagonist, S961, blocks the signal induced by both insulin and steviol glycosides. Upon the receptor activation, steviol glycoside signal leads to Glut4 translocation from intracellular pool to the plasma membrane, allowing glucose entry into the cell and thus mimicking the insulin action. Steviol glycosides are also able to increase the activity and the expression of the antioxidant enzymes SOD and CAT, probably through the activation of the same pathway (dashed blue lines). Moreover, cytosolic GSH levels are significantly increased in rat cardiac fibroblasts treated with steviol glycoside mixtures.

In conclusion, steviol glycosides exert pleiotropic effects on rat cardiac fibroblasts due to their ability to modulate the signaling pathways involved in glucose uptake and upregulation of the endogenous antioxidant defence system.  Figure 11 summarizes the obtained results.

These results suggest a potential role of *Stevia rebaudiana* not only as an antihyperglycaemic agent but also as a powerful cardio protective tool in cardiac fibroblasts that have been recently defined as “the renaissance cells” [54] due to their fundamental role in maintaining cardiac function.

## Abbreviations

IR: Insulin receptor
IGF-1: Insulin growth factor-like 1
IGF-1R: Insulin growth factor-like 1 receptor
AMPK: AMP-activated protein kinase.
